# Should Not Safeguards from Venereal Disease Be Thrown around Marriage?*Read before the Society for Sanitary and Moral Prophylaxis, April 12, 1906.

**Published:** 1906-11

**Authors:** John A. Wyeth

**Affiliations:** New York


### Abstracts and Selections

### Should Not Safeguards From Venereal Disease be Thrown Around Marriage?*

BY JOHN A. WYETH, M. D., NEW YORK.

To the question to which I have been invited to speak, namely, "In view of the grave injury to the family and race from venereal infection, should not safeguards be thrown around marriage?" there can be but one answer and that in the affirmative.

The chief difficulty lies in determining the better method of securing this protection, especially to the woman and her offspring.

In my opinion, in discussing this important feature of the subject we can eliminate that large proportion of the gentler sex who appreciate the possibilities of maternity and are looking forward under both of the natural and the social law to the enjoyment of this privilege.

From the dangers of direct infection before marriage this higher type of woman is protected not only by her innate purity, but by the

*Read before the Society for Sanitary and Moral Prophylaxis, April 12, 1906.

safeguards of her home and family and the rulings of modern civilization.

I would suggest that the solution of this problem can be best accomplished by dealing directly with the male side of the question, both married and unmarried, and we need not hesitate to speak plainly with him.

There is not a physician of experience who does not appreciate the importance of this subject. There can be few who are not ready and willing to contribute to the best of their ability to the proper dissemination of that knowledge which alone will lead to the diminution of the spread of these infectious diseases.

That the public knows practically nothing of the subject is a reflection upon our profession in its failure to inform them, and upon us should rest the blame if they fail to realize the gravity of the dangers which threaten not only society but public health.

Can one doubt for a moment that if mankind were aware of the fact that 90 per cent of all cases of locomotor ataxia and most all of the paralytic attacks, that 80 per cent of all the deaths from inflammatory diseases peculiar to women, and at least 50 per cent of all the operations known in gynecology, as well as 30 per cent of all the blindness in infancy and children were due to these diseases—I say again, can one doubt for a moment that with this knowledge in mind the public would not take steps to lessen the possibilities of these infections?

In our effort to safeguard marriage we must educate our men, and especially our younger men, to know that immoral association will in at least 95 per cent of cases result in the contraction of a loathsome disease which, in countless instances, may be seemingly cured for years and then break out again with deplorable consequences not only to the individual, but to innocent persons.

The chief educational agent of this age is the public press, and this society, with its high aims in view and with the strong influence of its membership, should labor earnestly to enlist its powerful aid in this work.

In the New York *Sun* of this date (April 12, 1906) there is an editorial entitled, "Morality and the Public Health," which calls attention to the very great dangers to the public health, to the innocent as well as the guilty, from the venereal infections, and there is paid this tribute to this organization:

"There is in New York City an organization known as the Society of Sanitary and Moral Prophylaxis, which has undertaken the delicate and difficult task of enlightening the public upon this important subject. It is entitled to the moral support of all persons who have at heart the welfare of mankind."—*New York State Journal of Medicine.*

